# Who is Getting the Message? Sociodemographic Factors and Gambling Behavior Associated with Awareness of the Swedish National Self-Exclusion Registry Spelpaus.se

**DOI:** 10.1007/s10899-024-10357-9

**Published:** 2024-10-07

**Authors:** Katya González Díaz, Magdalena Lagerlund, Mikael Rostila, Jenny Cisneros Örnberg

**Affiliations:** 1 Department of Public Health Sciences, Stockholm University, Stockholm, Sweden; 2 Swedish National Institute of Public Health, Stockholm, Sweden

**Keywords:** Self-exclusion, Awareness, Gambling, Sweden, Sociodemographic factors, PGSI

## Abstract

Awareness and utilization of health interventions can be influenced by sociodemographic factors. These factors play a role in information processing, particularly regarding health messaging. The aim of this study is to analyze how different sociodemographic factors and gambling behaviors are associated with awareness of a (Swedish) gambling self-exclusion register. The study used data from the Swedish longitudinal gambling study (Swelogs) and analyzed n = 6720 participants from a stratified random sample of the Swedish population aged 18–84 years. Logistic regressions were conducted to analyze associations between sociodemographic characteristics, gambling behavior and awareness. Males with middle (OR = 1.70, 95% CI = 1.32–2.19) and high income (OR = 1.58, 95% CI = 1.19–2.09) and females with high (OR = 1.35, 95% CI = 1.03–1.76) and middle-level education (OR = 1.35, CI = 1.04–1.76), had higher awareness of Spelpaus.se. Online gamblers with problematic gambling behavior were three times (OR = 3.69, 95% CI = 2.15–6.37) more likely to be aware than non-gamblers. Online gamblers born in Sweden were twice (OR = 1.95, 95% CI = 1.19–3.19) as likely to be aware compared to those born outside of Europe. Males with high level of awareness had higher PGSI scores compared to women. Education could increase awareness of potential health risks and opportunities for intervention, particularly among women. Future interventions should be tailored to cater to the specific needs of individuals with lower levels of awareness, to facilitate enhancing their awareness and ultimately promoting a more equitable utilization of self-exclusion measures.

## Introduction

Harmful gambling is widely recognized as a public health issue that affects individuals, families, and communities, encompassing social, economic, and health aspects (Hilbrecht et al., 2020; Marionneau et al., 2023; Wardle et al., 2021). Health inequalities related to gambling harms mean that certain individuals are more affected than others based on their specific social, demographic, and environmental conditions, even when exposed to comparable levels of gambling (Raybould et al., 2021). Ongoing efforts to reduce public health burden while protecting at-risk gamblers have been suggested to focus on minimizing gambling-related harms. Self-exclusion serves as an important intervention tool in achieving this goal (Blank et al., 2021; Blaszczynski et al., 2007; Gainsbury, 2014; Matheson et al., 2019; Wardle et al., 2018).

Self-exclusion is considered a responsible gambling measure that usually refers to the possibility of voluntarily suspending oneself from gambling for a predetermined period of time with one or more gambling companies. Spelpaus.se, the Swedish self-exclusion register, was introduced on January 1st, 2019, in connection with a re-regulation of the Swedish gambling market. According to the Gambling Act, SFS (2018:1138), individuals who self-exclude via Spelpaus.se will be prevented from gambling with any licensed gambling company in Sweden, whether land-based or online, for a period of 1, 3, or 6 months, or until further notice based on their preference. Licensed gambling companies operating under Swedish regulation need to provide information about Spelpaus.se in their advertising, encourage responsible gambling and provide information about where to seek help. Spelpaus.se messaging is provided on gambling products, and posters at gambling venues, in the form of public service announcements, on interactive terminals in gambling venues, and on government and licensed gambling provider websites.

Previous studies have highlighted the benefits of self-exclusion, including its overall effectiveness (Hing et al., 2015), protection against excessive gambling (Caillon et al., 2019), reduced frequency and expenditure on gambling (Kotter et al., 2019), improved financial situations (Luquiens et al., 2019), fewer negative impacts, including psychological effects (Tremblay et al., 2008), decreased urges to gamble (Townshend, 2007), and a greater sense of control over gambling (Hayer & Meyer, 2010, 2011; Ladouceur et al., 2007; Pickering et al., 2018). However, despite these advantages, research indicates that self-exclusion programs are underutilized by individuals with gambling problems (Gainsbury et al., 2020).

Studies suggest that the low utilization of self-exclusion programs may be related to a lack of awareness about their existence (Devault-Tousignant et al., 2023; Motka et al., 2018; Nowatzki & Williams, 2002). A systematic review conducted by Bijker et al. (2023) estimated that about 11% of people who gamble are aware of self-exclusion as a harm reduction tool. Similarly, a study by Håkansson and Henzel (2020) on self-exclusion in Sweden found that one of the reasons individuals with problematic gambling behavior or a higher risk of gambling problems do not utilize the national self-exclusion registry, Spelpaus.se, is because they are unaware of its existence.

The process of helping individuals make risk-reducing behavioral changes begins with awareness, which is considered a key component in various health behavior theories (see for example Glanz et al., 2015) and decision-making models. Rogers et al. (2014) define awareness as exposure to an idea including the initial reception and level of attention given to health information. Health literacy refers to an individual’s level of knowledge, motivation, and ability to access, understand, evaluate, and apply health information in their daily life, enabling them to make decisions about healthcare, disease prevention, and health promotion (Nutbeam & Lloyd, 2021; Sørensen et al., 2012). Individuals with low sociodemographic positions tend to have lower health literacy which can impact their awareness and engagement of health interventions (Beauchamp et al., 2015; Berkman et al., 2011; Van Der Heide et al., 2013). Accordingly, previous studies have shown associations between sociodemographic factors, such as economic level and educational attainment, and health information and literacy (Paasche-Orlow & Wolf, 2007; Svendsen et al., 2020) influencing information processing. Furthermore, increased awareness of self-exclusion programs is important to improve responsible gambling practices, and for self-exclusion to be effective as a protective measure (Hayer & Meyer, 2011; Louderback et al., 2023; Tong et al., 2019). However, the manner in which awareness of Spelpaus.se, as a harm reduction intervention, varies by sociodemographic factors and, especially, gambling behavior in relation to biological sex and gambling modality remains unknown. In this study, we aim to analyze how different sociodemographic factors and gambling behavior are associated with awareness of Spelpaus.se in men and women, as well as among land-based and online gamblers. The following research questions will be addressed:

1. What is the relationship between sociodemographic factors, gambling behavior and awareness of Spelpaus.se?
2. To what extent do associations between sociodemographic factors and awareness of Spelpaus.se vary by biological sex and gambling modality (land-based only vs. online gambling)?

## Methods

This study uses data from the Swedish Longitudinal Gambling Study (Swelogs), one of the world’s largest population-based studies examining the relationship between gambling and health. A new prevalence survey was conducted by the Swedish Public Health Agency in 2021 in a stratified random sample of people 16 to 84 years of age from the Register of the Total Population, with a response rate of 35% (Public Health Agency of Sweden, 2022).

### Participants

Participants in this study are respondents of the 2021 prevalence survey. A total of about 7,300 people responded from a random sample of 25,000 people.

#### Measures

*Awareness of Spelpaus.se* was measured with a survey question asking participants if they knew that via Spelpaus.se they could self-exclude from all gambling requiring registration with licensed gambling companies in Sweden. The response options were: “No”; “Yes, but haven’t used it”; and “Yes, and have used it” of which the latter two were combined to “Yes” due to limited response numbers. Before merging the two Yes responses regarding awareness, we evaluated their similarity by conducting a chi-square test. The test indicated that the responses were not statistically different from each other, suggesting that merging them would have limited impact on the findings.

*Sociodemographic factors* were measured with information from population-based registers and included; biological sex, age, education, income, family situation and country of birth. Biological sex was measured as a dichotomous variable (female or male). Age was categorized into four groups (18–24, 25–44, 45–64 and 65–84). Education and income were both divided into low, middle and high. Low education (ISCED codes 0–2) corresponds to elementary school, middle education (ISCED codes 3) to high school (or secondary school), and high level (ISCED codes 4 and higher) corresponds to post-secondary education. Income (individual disposable yearly income) was categorized into quartiles based on the whole sample. Low income equals the lowest quartile, up to 143,826 SEK (approx. 12,500 EUR) yearly, middle income equals the two middle quartiles, 143,827 SEK (approx. 12,500.01 EUR) to 337,904 SEK (approx. 29,400 EUR) and high income equals the highest quartile 337,905 SEK (approx. 29,400.01 EUR) or more. Family situation was categorized into four groups depending on whether the respondents were single or had spouses/cohabitants/partners (categorized in this study as “married”) and whether they had children living at home or not. Country of birth was categorized into three groups (Sweden, Europe and outside of Europe).

*Gambling behavior* was measured in Swelogs using the Problem Gambling Severity Index (PGSI) (Ferris & Wynne, 2001), a standardized measure of risk gambling behavior. In this study, gambling behavior is classified into three categories depending on PGSI score. Those with a PGSI score of 0 include both non-gamblers and those considered not-at-risk gamblers. A PGSI score of 1–2 was considered low-risk gambling. Finally, a PGSI score of 3 or higher was considered moderate risk or problem gambling.

*Gambling activity* categorized participants into three groups according to the amount and modality of gambling: “never”; “ever, but not in the past year”; “only land-based in past year” and “online in past year”.

*Gambling modality* was measured based on participants’ responses to gambling activity. Land-based gamblers did not gamble online during the past year, while online gamblers might have also gambled in the land-based modality.

#### Procedure

Data were collected by Statistics Sweden between August 27 and November 8, 2021. Invitations to participate were sent by mail with information about the study, a link to an online survey and with an offer of a phone interview as an alternative. A maximum of three reminders were mailed including an option to submit a paper version of the survey. Responding to the survey was regarded as informed consent to subsequently add individual data on age, biological sex and other sociodemographic factors from population registers using the unique national identification number assigned to each resident at birth or immigration.

#### Statistical Analysis

Survey weights were provided to statistically adjust the distribution of biological sex, age, sociodemographic factors, region of birth, education, income and estimated risk of gambling problems (based on sociodemographic factors from population registers) to that of the general population. In this study, weighted data were used for the descriptive analysis to reflect the prevalence in the population, whereas unweighted data were used for the logistic regression analysis and to examine associations. This was done to avoid the risk of single high weights in cells with low numbers skewing the results, potentially leading to the wrong conclusions. However, all significance tests were confirmed with weighted data. The sample distribution for the total sample and two sub-groups based on gambling modality is presented using weighted numbers and percentages. For comparison, unweighted numbers are also included for the total sample. Descriptive statistics for awareness of Spelpaus.se were calculated as proportions and 95% confidence intervals of the populations using weighted data. Logistic regression analysis was used with unweighted data to estimate odds ratios and 95% confidence intervals for awareness by sociodemographic factors, PGSI score and gambling activity. In addition to crude estimates, we also calculated estimates adjusted by age and biological sex, and further adjusted estimates by all sociodemographic factors. The full multivariate model included all sociodemographic factors as well as PGSI score and gambling activity. The full model was further stratified by biological sex and gambling modality. SPSS version 27 was used for the statistical analyses.

#### Ethics

The present study was approved by the Swedish Ethical Review Authority (Number 2020–00451). Participants were informed about the surveys research purpose and data protection legislation.

## Results

Our study sample consisted of 6720 participants aged 18 to 84 years. The sociodemographic distribution of the total sample, as well as among land-based gamblers and online gamblers, is presented in Table 1. There was an even biological sex distribution in the total sample, and the mean age was 48.9 years. Middle-level education was most prevalent (42.8%), followed by high level (36.4%). Most were married without children living at home (35.3%) or single without children living at home (31.8%) and born in Sweden (77.5%). Also, the majority had a PGSI score indicating that they were non-gamblers or not-at-risk gamblers (95.7%). When looking at those who had gambled in the past 12 months, land-based gamblers were older than online gamblers (mean age: 55.4 vs. 48.7 years), had a lower mean income (261.6 vs. 304.5) and a higher proportion of females (56.1% vs. 48.7%). Among online gamblers 4.1% had a PGSI score of 3 or more, compared to 0.5% among land-based gamblers.

**Table 1:** Distribution of socio-demographic variables within the total adult population, those who gambled online in the past 12 months and those who did not gamble online in the past 12 months

	Total sample unweighted (n = 6720) n (%)	Total sample weighted (%)	Land-based gamblers in past 12 months weighted (n = 1188) n (%)	Online gamblers in past 12 months weighted (n = 1844) n (%)
Biological sex			Pearson *X*^2^ = 80.277 p-value ≤ 0.001	
Male	3122 (46.5)	50.2	43.9	60.1
Female	3598 (53.5)	49.5	56.1	39.9
Mean age (SD)	50.5 (20.2)	48.9 (18.2)	55.4 (17.3)	48.7 (16.6)
Age (years)			Pearson *X*^2^ = 111.962 p-value ≤ 0.001	
18–24	1232 (18.3)	10.0	4.9	6.8
25–44	1333 (19.8)	33.6	22.6	35.0
45–64	2112 (31.4)	32.7	38.2	38.9
65–85	2043 (30.4)	23.7	34.3	19.3
Education			Pearson *X*^2^ = 4.505 p-value ≤ 0.105	
Low	1362 (20.3)	18.5	19.0	16.8
Middle	2559 (38.1)	42.1	46.1	49.6
High	2609 (38.8)	36.4	33.7	32.2
*Missing*	*190 (2.8)*	*3.0*	*1.2*	*1.3*
Mean income (SD) in thousand SEK	260.7 (421.4)	271.8 (374.5)	261.6 (162.8)	304.5 (263.1)
Income			Pearson *X*^2^ = 29.838 p-value ≤ 0.001	
Low	1992 (29.6)	23.7	29.0	14.6
Middle	3051 (45.4)	50.2	57.4	53.4
High	1642 (24.4)	25.1	23.3	31.7
*Missing*	*35 (0.5)*	*1.1*	*0.3*	*0.4*
Family situation			Pearson *X*^2^ = 17.223 p-value ≤ 0.001	
Single without children	2720 (40.5)	31.8	27.2	27.1
Single with children	373 (5.6)	3.5	4.6	4.0
Married without children	2154 (32.1)	35.3	42.1	37.2
Married with children	1392 (20.7)	28.0	24.5	31.1
*Missing*	*81 (1.2)*	*1.3*	*1.7*	*0.6*
Country of birth			Pearson *X*^2^ = 14.460 p-value ≤ 0.001	
Sweden	5680 (84.5)	77.5	85.9	83.7
Rest of Europe	524 (7.8)	9.7	8.2	6.5
Outside of Europe	488 (7.3)	11.8	5.9	9.4
*Missing*	*28 (0.4)*	*1.0*	*0.1*	*0.4*
PGSI			Pearson *X*^2^ = 89.345 p-value ≤ 0.001	
0	6467 (96.2)	95.7	97.0	87.0
1–2	170 (2.5)	3.0	2.5	8.9
3 +	83 (1.2)	1.3	0.5	4.1
Gambling activity				
Never	2054 (30.6)	29.0	–	–
Ever, but not in the past year	1525 (22.7)	24.3	–	–
Only land-based in past year	1188 (17.7)	17.1	–	–
Online in past year	1844 (27.4)	29.6	–	–
*Missing*	109 (1.6)	*1.5*	–	–
Aware of Spelpaus.se			Pearson *X*^2^ = 47.862 p-value ≤ 0.001	
No	4469 (66.5)	67.3	65.6	54.9
Yes, but never used it	2075 (30.9)	31.5	34.4	42.4
Yes, and used it	69 (1.0)	1.2	0.5	2.8
*Missing*	*107 (1.6)*	*1.6*	*1.5*	*0.7*

A third of all respondents reported that they were aware of Spelpaus.se (32.7%), with a higher degree of awareness among online gamblers (45.2%) compared to land-based gamblers (34.9%). Table 2 shows the level of awareness across sociodemographic sub-groups and PGSI categories. Awareness was generally higher among males, with high income, born in Sweden, and with a PGSI score above 0. For land-based gamblers awareness was highest among those aged 18–44 and those married without children living at home. In the case of online gamblers, awareness was higher among those aged 25–44 and those single with children living at home.

**Table 2:** Awareness of Spelpaus.se among adults by sociodemographic factors and gambling behavior

	Proportion in the total sample % (95% CI)	Proportion among land-based gamblers in past 12 months % (95% CI)	Proportion among online gamblers in the past 12 months % (95% CI)	
All	32.7% (31.6%–33.8%)	34.4% (31.7%–37.1%)		45.1% (42.0%–47.3%)
Biological sex				
Male	37.6% (36.0%–39.2%)	39.0% (34.8%–43.2%)		51.7% (49.8%–54.5%)
Female	27.6% (26.1%–29.1%)	30.9% (27.4%–34.4%)		35.2% (31.9%–38.5%)
Age (years)				
18–24	26.7% (23.4%–30.0%)	40.7% (28.1%–53.2%)		43.4% (41.2%–45.5%)
25–44	34.6% (32.7%–36.5%)	39.0% (33.2%–44.8%)		46.6% (44.5%–48.8%)
45–64	35.0% (33.0%–36.9%)	32.5% (28.1%–36.8%)		45.7% (43.6%–47.9%)
65–85	29.3% (27.1%–31.5%)	32.5% (27.9%–37.1%)		41.9% (39.7%–44.0%)
Education				
Low	31.2% (28.7%–33.8%)	37.3% (30.9%–43.6%)		49.3% (47.1%–51.4%)
Middle	33.3% (31.6%–35.0%)	32.9% (29.0%–36.9%)		42.5% (40.3%–44.6%)
High	33.5% (31.7%–35–4%)	34.0% (29.4%–38.6%)		48.4% (46.2%–50.5%)
Income				
Low	24.9% (22.8%–27.0%)	34.2% (28.0%–40.5%)		40.7% (38.5%–42.8%)
Middle	33.7% (32.2%–35.3%)	34.9% (31.3%–38.5%)		44.3% (42.2%–46.5%)
High	38.3% (36.0%–40.5%)	33.1% (27.6%–38.6%)		49.1% (46.9%–51.2%)
Family situation				
Single without children	31.1% (29.2%–33.1%)	34.3% (29.1%–39.5%)		46.8% (44.6%–48.9%)
Single with children	34.7% (28.8%–40.6%)	20.8% (9.8%–31.7%)		54.3% (52.2%–56.5%)
Married without children	34.0% (32.1%–35.8%)	36.7% (32.4%–40.9%)		46.3% (44.2%–48.5%)
Married with children	32.8% (30.8%–34.9%)	32.0% (26.6%–37.3%)		41.3% (39.2%–43.4%)
Country of birth				
Sweden	36.1% (34.9%–37.4%)	36.2% (33.3%–39.2%)		46.4% (44.3%–48.6%)
Rest of Europe	20.9% (17.8%–24.0%)	18.1% (10.3%–25.9%)		37.3% (29.1%–45.5%)
Outside of Europe	20.6% (17.8%–23.3%)	30.4% (19.6%–41.3%)		40.5% (33.5%–47.5%)
PGSI				
0	31.3% (30.2%–32.4%)	35.2% (32.4%–37.9%)		41.5% (39.3%–43.6%)
1–2 low risk	59.0% (52.3%–65.6%)	10.7% (0%–22–2%)		65.9% (63.9%–68.0%)
3 + Moderate risk/problem gambling	72.2% (63.0%–81.5%)	16.7% (0%–46.5%)		77.1% (75.3%–78.9%)

Results of the logistic regression analyses of awareness by sociodemographic and gambling factors are presented in Table 3. The bivariate (crude) logistic regression analysis showed that awareness of Spelpaus.se was higher among males, those between the ages of 45–64 years, those with high income, those born in Sweden, those with a PGSI score of 3 + , and those who have gambled online in the past year. After adjusting for age and biological sex associations between education and awareness become weaker while the association with family type turns statistically non-significant. Comparing the non-adjusted model controlling for age and biological sex, with the fully adjusted model we can observe that age and education are no longer relevant in the final model.

**Table 3:** Logistic regression analysis for awareness of Spelpaus.se by sociodemographic and gambling factors in the total adult sample. n = Unweighted number of people included in the full model

	Crude OR	Adjusted OR (by age and biological sex)	Multivariate OR (adj. by all sociodem)	Multivariate OR (full) (n = 6283)
Biological sex				
Men	1.54 (1.39–1.71)	1.55 (1.39–1.72)	1.54 (1.38–1.71)	1.35 (1.21–1.51)
Women	Ref	Ref	Ref	Ref
Age (years)				
18–24	Ref	Ref	Ref	Ref
25–44	1.63 (1.37–1.93)	1.64 (1.38–1.95)	1.31 (1.05–1.49)	1.16 (0.92–1.46)
45–64	1.72 (1.47–2.01)	1.71 (1.46–2.01)	1.27 (1.03–1.57)	1.11 (0.89–1.38)
65–85	1.38 (1.18–1.62)	1.35 (1.15–1.59)	1.01 (0.83–1.24)	1.00 (0.81–1.23)
Education				
Low	Ref	Ref	Ref	Ref
Middle	1.46 (1.26–1.70)	1.29 (1.10–1.51)	1.14 (0.97–1.35)	1.10 (0.93–1.30)
High	1.45 (1.25–1.68)	1.28 (1.08–1.51)	1.14 (0.96–1.35)	1.16 (0.97–1.38)
Income				
Low	Ref	Ref	Ref	Ref
Middle	1.66 (1.46–1.89)	1.58 (1.36–1.83)	1.38 (1.18–1.62)	1.24 (1.06–1.46)
High	1.98 (1.71–2.28)	1.70 (1.43–2.02)	1.46 (1.21–1.75)	1.29 (1.07–1.56)
Family situation				
Single without children	Ref	Ref	Ref	Ref
Single with children	1.28 (1.02–1.61)	1.13 (0.89–1.43)	1.09 (0.85–1.39)	1.05 (0.81–1.35)
Married without children	1.29 (1.15–1.46)	1.15 (1.01–1.31)	1.09 (0.95–1.25)	1.08 (0.94–1.23)
Married with children	1.22 (1.06–1.41)	0.96 (0.81–1.12)	0.90 (0.76–1.06)	0.89 (0.75–1.05)
Country of birth				
Sweden	2.37 (1.86–3.02)	2.43 (1.91–3.11)	2.20 (1.70–2.86)	1.96 (1.49–2.59)
Rest of Europe	1.47 (1.08–2.01)	1.49 (1.09–2.05)	1.52 (1.09–2.13)	1.45 (1.03–2.05)
Outside of Europe	Ref	Ref	Ref	Ref
PGSI				
0	Ref	Ref	Ref	Ref
1–2 low risk	2.88 (2.12–3.93)	2.78 (2.03–3.81)	2.98 (2.16–4.13)	1.93 (1.39–2.69)
3 + Moderate risk/problem gambling	4.47 (2.81–7.10)	4.15 (2.60–6.63)	5.13 (3.12–8.43)	3.25 (1.97–5.35)
Gambling activity				
Never	Ref	Ref	Ref	Ref
Ever, but not in the past year	2.14 (1.83–2.50)	1.95 (1.66–2.62)	1.77 (1.50–2.09)	1.75 (1.48–2.06)
Only land-based in past year	2.27 (1.93–2.68)	2.16 (1.83–2.56)	1.92 (1.62–2.29)	1.86 (1.56–2.22)
Online in past year	3.77 (3.26–4.36)	3.37 (2.90–3.92)	3.06 (2.61–3.57)	2.74 (2.33–3.22)

According to the full multivariate model biological sex, income, country of birth, PGSI score and gambling activity were statistically significantly associated with awareness of Spelpaus.se. A stratified analysis was performed controlling for biological sex and gambling modality. Males showed a 35% higher likelihood of awareness than females (OR = 1.35, 95% CI [1.21, 1.51]) and those with the highest income had a 29% higher likelihood of being aware than those with the lowest income (OR = 1.29, 95% CI [1.07, 1.56]). Respondents born in Sweden were twice as likely to be aware of the self-exclusion registry compared to those born outside of Europe (OR = 1.96, 95% CI [1.49,2.59]). Moreover, those with a PGSI of 3 + were three times more likely to be aware of Spelpaus.se than those who do not gamble (OR = 3.25, 95% CI [1.97,5.35]). Finally, those who gambled online in the past year were almost three times as likely to be aware of Spelpaus.se than those who have never gambled (OR = 2.74, 95% CI [2.33, 3.22]).

The stratified analysis was performed after statistically significant interactions were found between biological sex and income, and biological sex and PGSI in the full multivariate model. Table 4 presents the full multivariate model stratified by biological sex and gambling modality (online or land-based). Results showed that biological sex had a somewhat stronger association with awareness among online gamblers compared to land-based gamblers. For males, those with middle (OR = 1.70) and high (OR = 1.58) income had higher awareness of Spelpaus.se than those with low income. Education had a statistically significant association with awareness among women, with higher odds among those with middle (OR = 1.35) and high education (OR = 1.35). Income was associated with awareness among males, but not among females. The only association between awareness and family situation was found among married women without children living at home (OR = 1.22). Women with PGSI-scores of 1–2 were found to be more aware than those not at-risk or non-gamblers, whereas for men highest awareness was found among those with PGSI-scores of 3 + . The effect of country of birth or PGSI was not statistically significant among land-based gamblers. For online gamblers odds of awareness were statistically significantly higher among those who are single (OR = 1.62).

**Table 4:** Stratified multivariate logistic regression (full model) analysis for awareness of Spelpaus.se by biological sex and gambling modality. n = Unweighted number of people included

	Multivariate OR (full) by biological sex		Multivariate OR (full) by land/online	
	Males (n = 2915)	Females (n = 3368)	Land-based only (n = 1142)	Online (n = 1803)
Biological sex				
Men	–	–	1.30 (1.01–1.69)	1.71 (1.41–2.10)
Women	–	–	Ref	Ref
Age (years)				
18–24	Ref	Ref	Ref	Ref
25–44	1.25 (0.87–1.69)	1.09 (0.79–1.50)	1.05 (0.58–1.91)	1.37 (0.89–2.11)
45–64	1.12 (0.91–1.54)	1.07 (0.79–1.45)	0.98 (0.58–1.66)	1.42 (0.95–2.15)
65–85	0.94 (0.69–1.29)	1.05 (0.79–1.39)	0.67 (0.40–1.11)	1.39 (0.92–2.09)
Education				
Low	Ref	Ref	Ref	Ref
Middle	0.94 (0.75–1.17)	1.35 (1.04–1.76)	0.85 (0.56–1.19)	1.09 (0.81–1.47)
High	1.06 (0.84–1.35)	1.35 (1.03–1.78)	0.89 (0.59–1.33)	1.29 (0.95–1.77)
Income				
Low	Ref	Ref	Ref	Ref
Middle	1.70 (1.32–2.19)	0.99 (0.80–1.23)	1.25 (0.86–1.80)	1.08 (0.79–1.47)
High	1.58 (1.19–2.09)	1.13 (0.86–1.48)	1.15 (0.74–1.78)	1.11 (0.79–1.56)
Family situation				
Single without children	Ref	Ref	Ref	Ref
Single with children	1.35 (0.88–2.04)	0.92 (0.66–1.28)	0.65 (0.35–1.08)	1.62 (1.06–2.48)
Married without children	0.90 (0.74–1.10)	1.22 (1.01–1.47)	1.27 (0.94–1.71)	0.96 (0.75–1.22)
Married with children	0.89 (0.69–1.14)	0.87 (0.69–1.11)	0.80 (0.54–1.20)	0.76 (0.58–1.01)
Country of birth				
Sweden	2.03 (1.365–3.01)	1.94 (1.32–2.87)	1.46 (0.66–3.23)	1.95 (1.19–3.19)
Rest of Europe	1.67 (1.02–2.75)	1.25 (0.77–2.06)	0.98 (0.38–2.56)	1.80 (0.96–3.39)
Outside of Europe	Ref	Ref	Ref	Ref
PGSI				
0	Ref	Ref	Ref	Ref
1–2 low risk	1.77 (1.19–2.65)	2.35 (1.29–4.29)	0.54 (0.17–1.67)	2.30 (1.59–3.33)
3 + Moderate risk/problem gambling	5.36 (2.62–10.96)	1.45 (0.64–3.28)	1.31 (0.20–8.41)	3.69 (2.15–6.37)
Gambling activity				
Never	Ref	Ref	–	–
Ever, but not in the past year	1.49 (1.17–1.91)	2.06 (1.64–2.58)	–	–
Only land-based in past year	1.76 (1.34–2.33)	1.94 (1.54–2.44)	–	–
Online in past year	2.85 (2.25–3.61)	2.56 (2.04–3.21)	–	–

## Discussion

Low utilization of self-exclusion programs by individuals with increased risk of gambling problems can be attributed to a lack of awareness of their existence. This study explored awareness of the Swedish national self-exclusion register and analyzed the associations between various socio-demographic factors and awareness. We also performed a stratified analysis on whether these associations varied according to biological sex and gambling modality. Overall, one-third of the respondents were aware of Spelpaus.se. Most online gamblers were male while the majority of land-based gamblers were female. Sociodemographic factors were found to be associated with awareness of Spelpaus.se, with individuals born in Sweden being twice as likely to be aware of Spelpaus.se compared to those born outside of Europe. Additionally, gambling behavior was linked to awareness, as those who engaged in online gambling and those with a PGSI score of 3 + (indicating at-risk or problematic gambling behavior), were three times more likely to be aware of Spelpaus.se than non-gamblers. Furthermore, online gamblers displayed a higher level of awareness compared to land-based gamblers. Moreover, women with middle and high education levels, as well as men with middle and high income showed higher levels of awareness.

Awareness of Spelpaus.se was higher among land-based gamblers who were married without children and among online gamblers who were single with children. These results are compatible with previous findings where online gamblers tended to be single, separated, or divorced (Gainsbury et al., 2015; Mora-Salgueiro et al., 2021), while land-based gamblers were usually married (Edgren et al., 2017). Prior studies have noted the importance of social ties, particularly marriage/cohabitation or having children living at home, in exerting social control over health behaviors and effectively discouraging risky or negative behavior (Sampson et al., 2006; Syvertsen, et al., 2023). Additionally, these social ties provide greater accessibility to health information and literacy, enabling gamblers to be more aware of health interventions (Drosatos et al., 2020; Lee et al., 2004; Sørensen et al., 2012).

Our findings show that most online gamblers were male while most land-based gamblers were female, corroborating findings of previous research (Gainsbury et al., 2015) and those of a previous Swedish study by Håkansson and Henzel (2020). A potential explanation for this is that men typically gravitate towards strategic games (Merkouris et al., 2016; Wardle et al., 2011), commonly played individually online while women prefer non-strategic forms of gambling, which are typically land-based and more social, such as lotteries and scratch cards (McCormack et al., 2014; Raisamo et al., 2015). These preferences may be related to biological sex differences in attitudes, skills, and emotions in response to environmental and social stimuli (Fattore et al., 2014; McCormack et al., 2014).

Another important finding is that individuals born in Sweden were twice as likely to be aware of Spelpaus.se compared to those born outside of Europe. An in-depth explanation of this result is not easily explained since foreign-born immigrants in Sweden are a heterogenous group, not least in terms of income, education, and other sociodemographic factors (Rostila & Fritzell, 2014). However, previous research suggested that individuals born outside Europe may face psychosocial barriers in accessing health information and health care (Mona et al., 2021), as well as lower health literacy and a lack of language proficiency (Beauchamp, et al., 2015). Moreover, foreign-born gamblers were found more likely to gamble on international websites without supervision from Swedish authorities or access to Spelpaus.se messaging (Swedish Gambling Authority, 2024).

A systematic review by Mora-Salgueiro and colleagues (2021) indicated that sociodemographic factors of gamblers differ depending on their gambling modality. Results of the review showed that problematic online gamblers were predominantly male and had higher incomes than those in the land-based variety. In accordance with this, our results also show that the association between gambling behavior and awareness differed by biological sex, with men frequently having a higher PGSI score than women. These differences could be attributed in part to the tradition of gambling being dominated by male gamblers, making them more exposed to available information regarding health promotion and health seeking related to gambling (McCormack et al., 2014; Svensson & Romild, 2014). On the other hand, we found that men who gamble online were more aware of Spelpaus.se than female online gamblers and that women with low-risk gambling behavior were more aware than non-gamblers or problematic gamblers. Women with low-risk gambling behavior seem to be perceptive to health-messaging and warnings, while those with problematic gambling behavior could ignore them by not seeing them as personally relevant (Kreuter et al., 1999; Tong et al., 2018).

Finally, our results demonstrate how education can contribute to an increased awareness of potential health risks and opportunities for intervention (Osborn et al., 2011; Paasche-Orlow & Wolf, 2007; Svendsen et al., 2020), particularly among women. Our findings align with previous studies that suggest women, particularly those with higher levels of education, tend to actively seek health-related information (Lee et al., 2021; Nölke et al., 2015), highlighting their openness to health messaging (Rockloff & Schofield, 2004). In contrast and consistent with the literature, awareness of the self-exclusion registry was more closely associated with income among men.

Male participants with middle and high income were more aware of Spelpaus.se compared to those with low income, which is compatible with previous findings regarding the connection between economic status and health information and literacy (Beauchamp et al., 2015; Berkman et al., 2011; Paasche-Orlow & Wolf, 2007; Raybould, et al., 2021; Svendsen et al., 2020; Van Der Heide et al., 2013). Consequently, individuals with lower incomes may encounter difficulties in accessing and comprehending information, including health messaging, leading to lower awareness of, for example, harm reduction tools.

Awareness of responsible gambling measures, such as self-exclusion via Spelpaus.se, is associated with sociodemographic characteristics and gambling modality. In general, online gamblers were clearly more aware of Spelpaus.se compared to those who only gambled land-based. Aside that, the association between awareness and other explanatory factors played out differently across the biological sexes. Increased awareness of the self-exclusion registry was, for example, linked to level of education among women, while male awareness was rather linked to level of income. The finding that males with high level of awareness were more likely to have higher PGSI scores compared to women, may suggest that awareness was in fact higher among those with the greatest risk of developing gambling related problems. On the other hand, it seems that individuals born outside of Europe were not as aware of Spelpaus.se as those born in Sweden. Future interventions should therefore be designed to address the specific needs of these groups.

### Limitations

There are some potential limitations to consider in our study. First, the non-response rate was relatively high, which could affect the generalizability of our findings. Specifically, if non-response is more prevalent among low socioeconomic status (SES) groups or migrants, it may lead to an underestimation of the association we observed. Another important factor to acknowledge is the possibility of residual confounding. For example, variables such as language proficiency or the number of years lived in Sweden could impact cultural integration among individuals born outside of Europe. Furthermore, we should take into account the way we categorized problematic gambling behavior. Specifically, merging moderate risk gamblers (PGSI 3 +) with problematic gamblers (PGSI 8 +) might have influenced our results, as problem gamblers are already experiencing negative consequences, whereas moderate risk gamblers may be at risk of developing problems. However, due to the small number of online problem gamblers in our sample, this groups could not be analyzed separately. Moreover, our study aims to assess awareness of self-exclusion messages among the general population, rather than only focusing on gamblers. We include non-gamblers as they may have varying levels of exposure to self-exclusion messages, which could affect their level of awareness. Furthermore, by including non-gamblers in our analysis, we can also consider the possibility of future gambling involvement or close relationships and connections with gamblers.

## Conclusion

Our study contributes to the existing research on the relationship between sociodemographic factors, gambling behaviors and awareness of self-exclusion programs. It highlights the importance of considering sociodemographic characteristics, especially biological sex, as well as gambling behavior and modality when designing gambling-related interventions aimed at harm reduction. Increasing awareness among vulnerable/exposed/at risk groups may also lead to behavioral changes and potentially higher use of tools such as Spelpaus.se. Further research should explore the various channels through which gamblers encounter health messages and how these messages are interpreted across different contexts. Additionally, it is important to understand the factors that influence whether individuals choose to act on these messages. We also recommend future studies to assess awareness levels among both non-users and users of Spelpaus.se.

## Data Availability

The data that support the findings of this study are not publicly available. The data are, however, available from the authors upon reasonable request and with the permission of the Public Health Agency of Sweden.
